# Digital Twin Neural Marker Discovery for Delineating Mixed Dementia with Cross‐site Federated Learning

**DOI:** 10.1002/alz70856_102548

**Published:** 2025-12-26

**Authors:** Byung‐Hoon Kim, Chang‐Bae Bang, Gyutaek Oh, Jinhak Kim, Manjae Kwon, Keun You Kim, Eosu Kim

**Affiliations:** ^1^ Yonsei University College of Medicine, Seoul, Korea, Republic of (South)

## Abstract

**Background:**

Mixed dementia is characterized by significant clinical and pathological heterogeneity posing challenges for accurate diagnosis and treatment planning. Digital twin technology offers a transformative approach by learning data characteristics to simulate predictions of an unseen data sample. This study aims to construct a digital twin neural marker with multimodal neuroimage data to enable predictive tasks on samples with mixed dementia. We leverage cross‐site neuroimage datasets from the Dementias Platform UK (DPUK) and a Korean dementia cohort to construct the digital twin neural marker and show that the marker can be applied to delineate etiologies of mixed dementia.

**Methods:**

A digital twin neural marker of mixed dementia was constructed by training a Vision Transformer (ViT) model with a generative pre‐training approach. Specifically, Masked AutoEncoder (MAE) training of the ViT model on T1w and FLAIR neuroimage data from DPUK and a Korean dementia cohort was first constructed to capture the latent characteristics of the neurodegenerative brain etiologies. The model was further fine‐tuned on another Korean dementia cohort data with mixed dementia to delineate the etiologies of the neurocognitive disorder given a neuroimage input. Federated learning approaches were employed to train the model without data transfer across sites, maintaining data privacy within the trusted research environment.

**Results:**

The fine‐tuned digital twin neural marker achieved a reliable performance in delineating the mixed dementia etiology. The latent representation of the neural marker showed a separable pattern between different underlying etiologies when visualized with UMAP. These findings demonstrate the effectiveness of digital twins in leveraging global cross‐site datasets to provide actionable clinical insights.

**Conclusions:**

The results highlight the potential of a digital twin neural marker to address the complexities of delineating mixed dementia etiologies. Most importantly, this work represents an international collaboration (Korea‐UK) to develop a digital twin neural marker using large datasets while upholding data democracy principles.

